# Current contrasting population trends among North American hummingbirds

**DOI:** 10.1038/s41598-021-97889-x

**Published:** 2021-09-15

**Authors:** Simon G. English, Christine A. Bishop, Scott Wilson, Adam C. Smith

**Affiliations:** 1 Environment and Climate Change Canada, Wildlife Research Division, Pacific Wildlife Research Centre, 5421 Robertson Road, Delta, BC V4K 3N2 Canada; 2 Environment and Climate Change Canada, Wildlife Research Division, National Wildlife Research Centre, 1125 Colonel By Drive, Ottawa, ON K1S 5B6 Canada

**Keywords:** Ecology, Statistical methods, Conservation biology

## Abstract

As pollinators, hummingbirds play a critical role for both the function of ecological communities and in providing ecosystem services for people. To examine the conservation status of North American hummingbirds, we analyzed Breeding Bird Survey data for 8 species and 3 genera from 1970 to 2019 (long-term) and from 2009 to 2019 (short-term, approximately three generations). Among the *Selasphorus* genus, Allen’s, rufous, and broad-tailed hummingbirds have declined since 1970, and the rate of decline increased from 2009 to 2019. Contrasting the trends from the past half-century, ruby-throated hummingbirds of Eastern North America have declined since approximately 2004 throughout most of the species’ breeding range. In contrast, Anna’s hummingbird populations have increased dramatically since 1970 in their range in western North America. We also tested whether apparent declines might be due to a growing mismatch between the timing of breeding and the timing of BBS surveys. We found no evidence for such an effect, thus supporting the hypothesis that trends reflect true demographic change. Our analyses and geographic modelling highlight the urgent need of regulatory action to conserve hummingbirds uniquely capable of filling their niche in North America.

## Introduction

We have reached a turning point in the modern era for biodiversity loss^1^. Human disturbance to habitat and climate change are the two greatest threats to biodiversity of our time^2,3^. Nearly ${60}{\%}$ of all bird species in North America are in decline, with a loss of almost ${30}{\%}$ of birds since 1970^4^ and others projected to become extinct within the next 30 years^5^. Evidently, the widespread risk of diversity loss is substantial, although resources allocated to conservation are not. Here we report on an underrepresented family of birds in the literature that may be at the forefront of these declines: the hummingbirds (*Trochilidae*).

Hummingbirds are an ecologically important family of birds in North America, pollinating at least 184 known plant species through co-evolution with the plants that provide nectar to fuel their high-energy flight^6–8^. Hummingbirds are among the most rapidly diversifying clades of birds in the world, due in part to the diversity of ecological niches they exploit^9^. As such, they possess diverse phenological and life-history characteristics. For instance, many of the North American hummingbirds are migratory^10^, a characteristic that makes them especially vulnerable to the impacts of climate change and human disturbance to habitat^11^. In contrast, urbanization and changing climate may have relieved environmental pressures for other species, particularly non-migratory hummingbirds that benefit from year-round access to introduced plant species and supplemental feeders^12,13^. Therefore, as human activity and climate change alter ecoregions differently^14,15^, hummingbird species may also be impacted at different rates.

Among the 14 species of North American hummingbirds that commonly occur north of Mexico^16^, some have experienced range transformations, while others have shown population declines since the 1970s when continental monitoring began with programs such as the North American Breeding Bird Survey (BBS)^10,13^. The family as a whole has experienced significant population declines^4^, although species including the ruby-throated hummingbird (*A. colubris*) and the Anna’s hummingbird (*C. anna*) have previously shown periods of population growth over the last 50 years^10,12^. The conservation status of hummingbird species must therefore be considered individually. Organisations like Partners in Flight and the Western Hummingbird Partnership actively promote hummingbird habitat conservation among declining western species^17,18^; however, detailed short-term data on population trends are needed to track the progress of these large-scale efforts throughout North America. We analyzed Breeding Bird Survey (BBS) data to examine range-wide and regional population trends for 8 species in 3 genera: calliope hummingbirds (*Selasphorus calliope*), broad-tailed hummingbirds (*Selasphorus platycercus*), rufous hummingbirds (*Selasphorus rufus*), Allen’s hummingbirds (*Selasphorus sasin*), black-chinned hummingbirds (*Archilochus alexandri*), ruby-throated hummingbirds (*Archilochus colubris*), Anna’s hummingbirds (*Calypte anna*), and Costa’s hummingbirds (*Calypte costae*). Species in the *Archilochus* and *Selasphorus* genera are medium-to-long-distance migrants with a resident subspecies of the Allen’s hummingbird expanding its range^13^. Both *Calypte* species are resident to short-distance migrants.

We analyzed the period from 1970 to 2019 and contrast these results with short-term analyses for the period from 2009 to 2019 to gain insight into population changes occurring over approximately three generations. Population-change over three generations is a commonly used time frame relevant to the life history of species to identify critically endangered, endangered, and threatened species^19^. Hummingbirds, like many North American birds, have begun to breed earlier in recent decades raising the possibility that apparent trends in long-term surveys like the BBS are actually due to an increasing mismatch between the timing of breeding and the timing of surveys. To test this hypothesis, we conducted an additional analysis that allowed us to examine the change in abundance while accounting for any shifts in breeding date across decades. Lack of support for this hypothesis would provide support for the alternative hypothesis that population trends reflect true demographic change. For this analysis, we selected a steeply declining species for which this mismatch has been hypothesized: the Allen’s hummingbird^13^. We focus on regional trends of each species to examine spatial variation in trends across the range of each species. Among the hummingbirds we analyzed, diverse life-history characteristics are represented, from resident species to long-distance migrants, urbanized species and species whose habitat is largely limited to shrubsteppe or forested landscapes, as well as species with diverse breeding phenologies. We consider how these different characteristics may relate to the population trends observed in our dataset.

## Results

**Figure 1:**
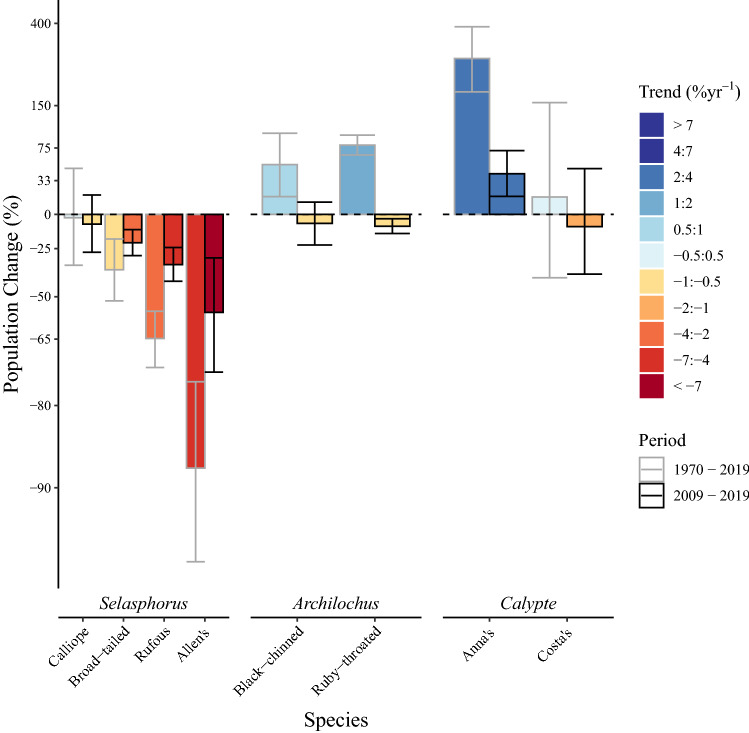
Percent change in populations of calliope hummingbirds (*Selasphorus calliope*), broad-tailed hummingbirds (*Selasphorus platycercus*), rufous hummingbirds (*Selasphorus rufus*), Allen’s hummingbirds (*Selasphorus sasin*), black-chinned hummingbirds (*Archilochus alexandri*), ruby-throated hummingbirds (*Archilochus colubris*), Anna’s hummingbirds (*Calypte anna*), and Costa’s hummingbirds (*Calypte costae*) across North America. Vertical axes scale to symmetry on the log-scale to accurately represent the percent change necessary for a population to recover to initial size at the beginning of the period. Column colours scaled to rate of change where darker hues reflect a greater rate of change. Errors shown are the upper and lower bounds of 90% credible-intervals.

We modelled hummingbird populations in North America from BBS data from 1970 to 2019 for long-term population trends and from 2009 to 2019 for short-term trends (Fig. 1). We analyzed 8 species in 3 genera for which there was sufficient BBS coverage, where $n_{lt}$ is the number of BBS routes included in long-term analyses and $n_{st}$ is the number of BBS routes included in short-term analyses: calliope hummingbirds (*Selasphorus calliope*; $n_{lt} = 223$; $n_{st} = 193$), broad-tailed hummingbirds (*Selasphorus platycercus*; $n_{lt} = 303$; $n_{st} = 267$), rufous hummingbirds (*Selasphorus rufus*; $n_{lt} = 408$; $n_{st} = 348$), and Allen’s hummingbirds (*Selasphorus sasin*; $n_{lt} = 59$; $n_{st} = 50$), black-chinned hummingbirds (*Archilochus alexandri*; $n_{lt} = 471$; $n_{st} = 399$), ruby-throated hummingbirds (*Archilochus colubris*; $n_{lt} = 2469$; $n_{st} = 2123$), Anna’s hummingbirds (*Calypte anna*; $n_{lt} = 266$; $n_{st} = 223$), Costa’s hummingbirds (*Calypte costae*; $n_{lt} = 97$; $n_{st} = 73$).

**Figure 2:**
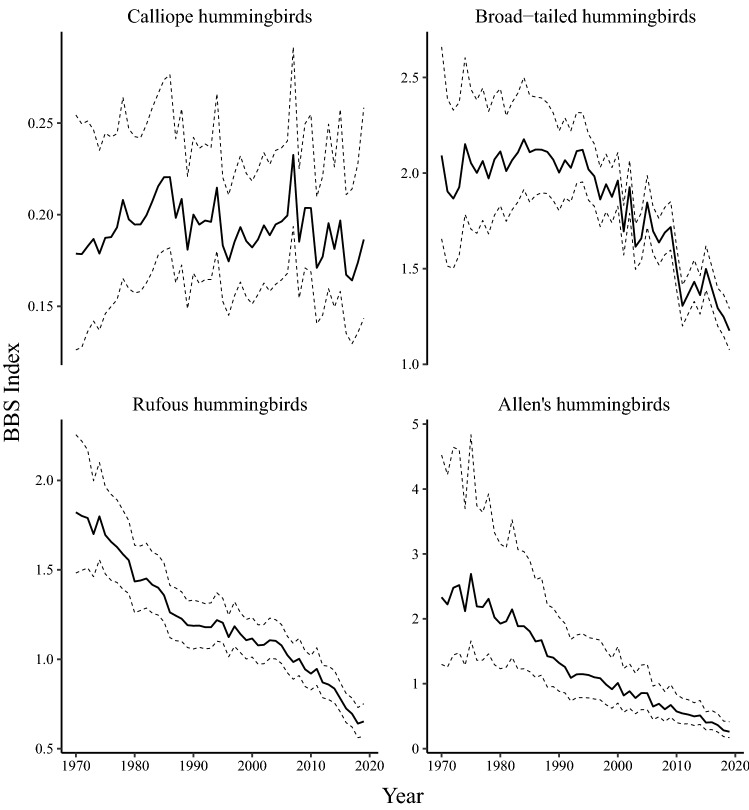
Breeding Bird Survey indices for North American hummingbirds in the *Selasphorus* genus from 1970 to 2019. The species of *Selasphorus* genus represented here are calliope hummingbirds (*Selasphorus calliope*), broad-tailed hummingbirds (*Selasphorus platycercus*), rufous hummingbirds (*Selasphorus rufus*), and Allen’s hummingbirds (*Selasphorus sasin*). Dashed lines represent upper and lower bounds of the ${90}{\%}$ credible-interval.

Three of the four species in the *Selasphorus* genus have declined since 1970, while the fourth species, the calliope hummingbird, has remained relatively stable (Fig. 2). From 1970 to 2019, Allen’s hummingbirds changed by ${-88}{\%}$ (CI ${-95} \,\text{to}\, {-76}{\%}$) at an average annual rate of ${-4.3}{\% \; \text{year}^{-1}}$ (CI $-5.8 \,\text{to}\, {-2.8\% \; \text{year}^{-1}}$) (Fig. 1). Allen’s hummingbirds suffered a dramatically steepened short-term decline at nearly a two-fold greater rate than over the past 50 years (${-7.9}{\% \; \text{year}^{-1}}$; CI $-12.4 \,\text{to}\, {-3.6}{\% \; \text{year}^{-1}}$) throughout regions of their range with sufficient coverage to estimate trends (Supplementary Fig. S1). From 1970 to 2019, rufous hummingbird net population change was ${-65}{\%}$ (CI ${-72} \,\text{to}\, {-56}{\%}$) at ${-2.1}{\% \; \text{year}^{-1}}$ (CI $-2.6 \,\text{to}\, {-1.7}{\% \; \text{year}^{-1}}$) and this rate of decline has also accelerated by nearly two-fold to ${-4.1}{\% \; \text{year}^{-1}}$ (CI $-5.5 \,\text{to}\, {-2.7}{\% \; \text{year}^{-1}}$) (Fig. 1). These trends appear to be most dramatic on the Pacific Coast of the rufous hummingbird’s range (Supplementary Fig. S2). Trend analyses of broad-tailed hummingbirds revealed a net population change of ${-37}{\%}$ (CI ${-52} \,\text{to}\, {-19}{\%}$) at ${-0.95}{\% \; \text{year}^{-1}}$ (CI $-1.5 \,\text{to}\, {-0.42}{\% \; \text{year}^{-1}}$) in the long-term, and ${-2.4}{\% \; \text{year}^{-1}}$ (CI $-3.4 \,\text{to}\, {-1.3}{\% \; \text{year}^{-1}}$) in the short-term (Fig. 1). Declines appear most significant in the southern extent of their range (Supplementary Fig. S3). Calliope populations are not changing significantly, either in the long-term (${-0.057}{\% \; \text{year}^{-1}}$; CI $-0.87 \,\text{to}\, {0.80}{\% \; \text{year}^{-1}}$) or the short-term (${-0.82}{\% \; \text{year}^{-1}}$; CI $-3.1 \,\text{to}\, {1.6}{\% \; \text{year}^{-1}}$) (Supplementary Fig. S5).

**Figure 3:**
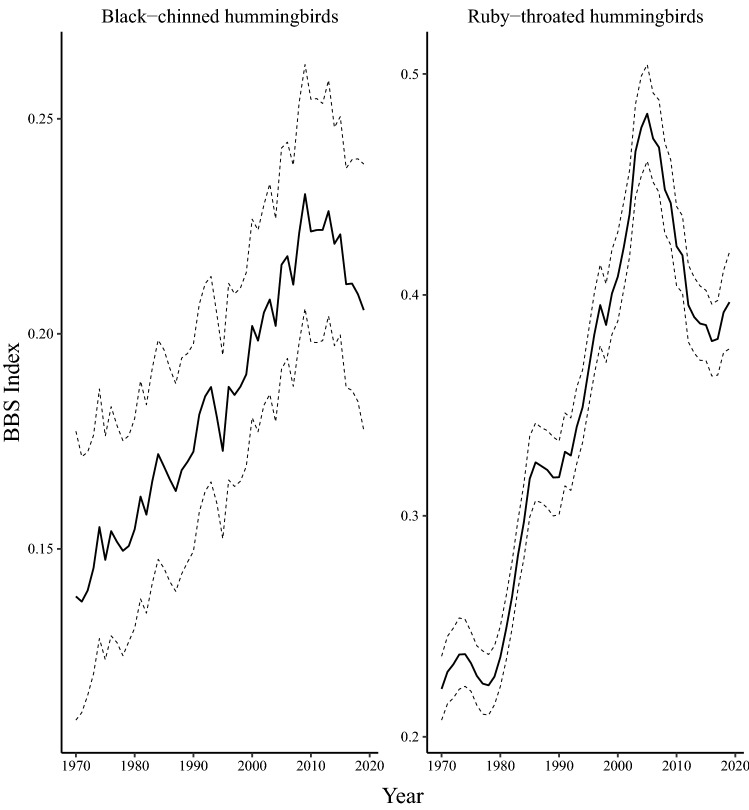
Breeding Bird Survey indices for North American hummingbirds in the *Archilochus* genus from 1970 to 2019. The species of *Archilochus* genus represented here are black-chinned hummingbirds (*Archilochus alexandri*) and ruby-throated hummingbirds (*Archilochus colubris*). Dashed lines represent upper and lower bounds of the ${90}{\%}$ credible-interval.

Continent-wide populations of both species in the *Archilochus* genus show increases over the long-term but declines from 2009 to 2019. From 1970 to 2019, the continent-wide black-chinned hummingbird population increased by ${52}{\%}$ (CI ${16} \,\text{to}\, {98}{\%}$) at a rate of ${0.86}{\% \; \text{year}^{-1}}$ (CI $0.31 \,\text{to}\, {1.40}{\% \; \text{year}^{-1}}$). Ruby-throated hummingbird population increased by ${79}{\%}$ (CI ${65} \,\text{to}\, {95}{\%}$) at a rate of ${1.2}{\% \; \text{year}^{-1}}$ (CI $1.0 \,\text{to}\, {1.4}{\% \; \text{year}^{-1}}$) since 1970 (Fig. 1). The short-term population trends from 2009 to 2019 were relatively stable for black-chinned hummingbirds (${-0.75}{\% \; \text{year}^{-1}}$; CI $-2.5 \,\text{to}\, {1.0}{\% \; \text{year}^{-1}}$) although there appears to be a signal of moderate declines throughout most of the species range (Supplementary Fig. S5). Trends also reversed for ruby-throated hummingbirds, where over a 10-year period, the continent-wide population changed by ${-9.5}{\%}$ (CI ${-15} \,\text{to}\, {-3.6}{\%}$) at an average rate of ${-0.99}{\% \; \text{year}^{-1}}$ (CI $-1.6 \,\text{to}\, {-0.36}{\% \; \text{year}^{-1}}$) throughout the breeding range (Fig. 3, Supplementary Fig. S6). This trend is more dramatic when estimated over the full 15-year period of decline. The average annual rate of decline from 2004 to 2019, ruby-throated hummingbirds in North American changed by an estimated ${-17}{\%}$ (CI ${-22} \,\text{to}\, {-10}{\%}$) at an average annual rate of ${-1.20}{\% \; \text{year}^{-1}}$ (CI $-1.7\, \text{to} \,{-0.73}{\% \; \text{year}^{-1}}$.

**Figure 4:**
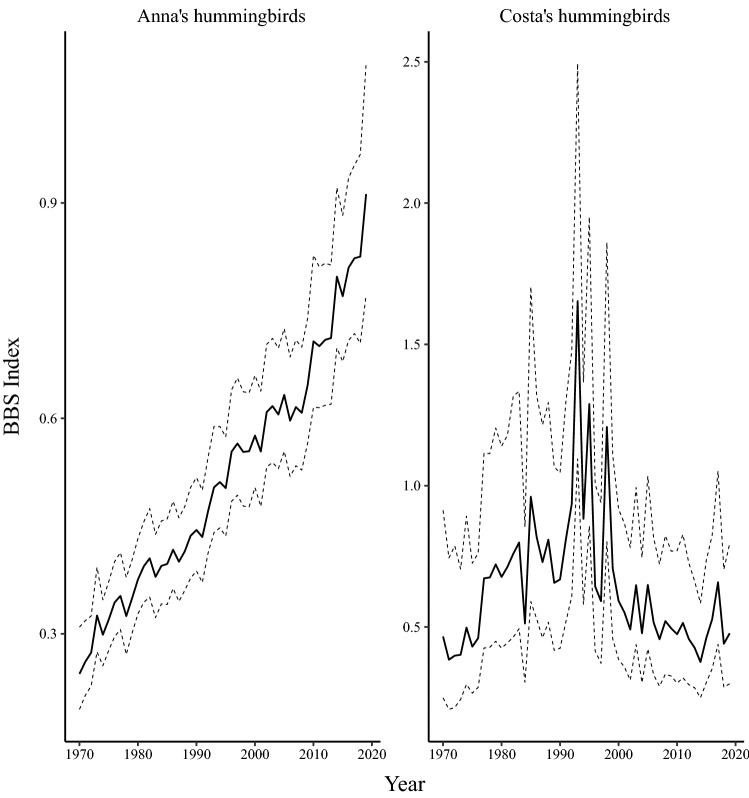
Breeding Bird Survey indices for North American hummingbirds in the *Calypte* genus from 1970 to 2019. The species of *Calypte* genus represented here are Anna’s hummingbirds (*Calypte anna*) and Costa’s hummingbirds (*Calypte costae*). Dashed lines represent upper and lower bounds of the ${90}{\%}$ credible-interval.

Continent-wide population levels of the *Calypte* genus have increased in the case of Anna’s Hummingbird or remained relatively stable in the case of Costa’s hummingbirds (Fig. 4). Anna’s hummingbirds increased from 1970 to 2019 at a rate of ${2.7}{\% \; \text{year}^{-1}}$ (CI $2.1 \,\text{to}\, {3.3}{\% \; \text{year}^{-1}}$). This trend steepened in the short-term (${3.5}{\% \; \text{year}^{-1}}$; CI $1.5 \,\text{to}\, {5.5}{\% \; \text{year}^{-1}}$), and was most dramatic in the northern and western extent of the species’ current range (Supplementary Fig. S7). Continent-wide Costa’s hummingbird populations did not change significantly in our long-term (${0.30}{\% \; \text{year}^{-1}}$; CI $-1.1 \,\text{to}\, {1.9}{\% \; \text{year}^{-1}}$) or short-term (${-1.0}{\% \; \text{year}^{-1}}$; CI $-4.9 \,\text{to}\, {3.9}{\% \; \text{year}^{-1}}$) trend analyses. Uncertainty on Costa’s hummingbird trend analyses are relatively large (Fig. 1), and may impact our ability to detect a significant signal of population decline reflected across the majority of their range (Supplementary Fig. S8).

To test the possibility of a phenological shift accounting for the declines observed in Allen’s hummingbird populations, we fit a modified generalized additive model with year effects (GAMYE) and included a parameter for date-of-observations within the hierarchical GAMYE structure. The date-of-observations effect was estimated for each decade of observations within the data to account for a potential phenological shift causing low species counts. We did not find evidence of survey date on the mean number of birds recorded across decades (Supplementary Figs.  S10, S11). Our long-term trend estimate for Allen’s hummingbirds corrected for phenological shift (${-4.1}{\% \; \text{year}^{-1}}$; $CI_{95\%}$ $-5.9 \,\text{to}\, {-2.3}{\% \; \text{year}^{-1}}$) was not significantly different from estimates derived using the standard GAMYE model (${-4.3}{\% \; \text{year}^{-1}}$; $CI_{95\%}$ $-6.0 \,\text{to}\, {-2.6}{\% \; \text{year}^{-1}}$).

## Discussion

By conducting an assessment of long-term and short-term population trends for the eight most abundant species among North American hummingbirds, we show the contrasting population trajectories in a critical step towards understanding threats and potential conservation approaches for this ecological group. Our analyses revealed alarming population declines in the *Selasphorus* genus. Allen’s, Rufous, and broad-tailed hummingbird declines have all accelerated over the last three generations to nearly double the rate of decline over the previous half-century. We also report previously undocumented declines of Eastern North America’s most common hummingbird: the ruby-throated hummingbird (Fig. 3), where over a 15-year period, the continent-wide population decreased by ${17}{\%}$ (Fig. 1). This short-term decline contrasts the species’ average population growth rate from 1970 to 2019 (Fig. 1). In contrast, the Anna’s hummingbird, a resident hummingbird in Western North America, has experienced dramatic population growth at an accelerating rate in the last decade (Fig. 1). Anna’s hummingbird populations have increased significantly in the USA but most extremely in the expanding northern edge of their range in Canada (Supplementary Fig. S7), where the population increased at a rate of ${20}{\% \; \text{year}^{-1}}$. The species was formerly rare in southern Canada even in the late 2000s but is now an abundant year-round resident in the Georgia Basin Ecosystem and commonly occurs as far north as southern Alaska.

The *Selasphorus* genus comprises medium-to-long distance migrants, including a resident population of Allen’s hummingbirds in residential parts of California with a reportedly stable or perhaps growing population^13^. Our data collected during the breeding season indicate similar declines in Canada and the USA for species which exist in both regions, both in the long-term and the short-term. While there is potential for a mismatch in breeding phenology, and thus detection, and surveying efforts^20,21^, an advancement of mean arrival date of several days is not likely to fully account for the drastic declines observed from northern and southern range limits even in our short-term analyses (Supplementary Fig. S2B), where Allen’s hummingbirds lost ${56}{\%}$ (CI 31–73%) of their population, while rufous hummingbirds lost ${35}{\%}$ (CI 24–43%) since 2009 (Fig. 1). We validated our conclusion that the survey trends reflect a true demographic change using a modified hierarchical generalized additive model to correct for under-counted observations due to misalignment between the breeding season and survey dates. This model includes an additional hierarchical structure that estimates the shift in timing of peak numbers of observations by decade. A shifting peak in observations outside of the range of permissible survey dates since the beginning of the BBS monitoring program would suggest under-counting of breeding adults. After correcting for potentially under-counted individuals due to misalignment of the breeding season and survey dates, means of annual trend estimates were not statistically significantly different. Still, the causes of population declines for migratory Allen’s, rufous, and broad-tailed hummingbirds are largely unknown.

For migratory hummingbirds, plant phenology may provide cues on the quality of stopover sites^22^. A mismatch between hummingbird migration and plant phenology could contribute to decline in hummingbird populations, which are exacerbated among migratory species^23^. This kind of phenological mismatch is thought to occur for migratory rufous hummingbirds^24^. Furthermore, as natural habitats are converted into urban environments, invasive species may impact the quality of stopover sites and breeding grounds in remaining non-urban environments. An overabundance of deer can impact ecosystems through sustained overbrowsing of herbaceous cover and flowers^25^, thus precipitating detrimental changes to the woodlot and meadow habitats^26,27^. High-density white-tailed deer populations in wooded habitats in North America significantly reduced species richness and abundance of intermediate canopy nesting songbirds^27^, where most hummingbirds build their nests. Additionally, chronic, widespread exposure to agrochemicals on breeding grounds may impact migratory hummingbirds^28–30^. Further study into the population-level effects of agrochemical exposure and invasive species in breeding grounds are warranted.

On the wintering grounds, the rufous hummingbird has reportedly experienced an expansion of wintering distribution, perhaps attributable to an increase in resources provided in gardens and genetic reprogramming of migratory orientation^31^. The authors acknowledge the potential for observer biases, wherein more resources for identification of rufous hummingbirds promotes more frequent reporting as well as an unknown increase in supplemental resource provisioning^31^. This highlights an advantage of BBS analyses for modelling population trends, because the data are systematically collected by skilled observers and are modelled with first-year-observer effects and inter-observer variability contributing to uncertainty estimates^32^. Observed declines for disparate populations in North America might also reflect threats on their non-breeding grounds^33^. For example, the wintering range of rufous, broad-tailed, and migratory Allen’s populations all include montane forests in central Mexico where there is both recent and future projected habitat loss^34^. Threats to *Selasphorus* hummingbirds are still poorly known and future research to identify those threats and where they are most effective should be considered a high priority.

The *Archilochus* hummingbird genus of medium-to-long distance migrants generally found at low elevation experienced population growth until approximately 2004. Trend maps for black-chinned hummingbirds from 2009 to 2019 reveal relatively stable or declining populations throughout most of the mapped region (Supplementary Fig. S5B). Ruby-throated hummingbirds show this same pattern, though with stronger declines over the last three generations (Supplementary Fig. S6B). Ruby-throated hummingbirds winter in dry tropical forests^17^, and although data on this wintering habitat is largely unavailable, Central American dry forests experienced substantial anthropogenic disturbance from 2000 to 2012^35^, suggesting that habitat loss may contribute to recent population declines. Moreover, the frequency and severity of forest fires in dry forest habitats increased significantly within the last two decades and this trend is projected to continue as climate-change further alters ecosystems^36^. Forest fires pose a considerable threat to the productivity and species richness of tropical dry forests^37^, highlighting another risk to hummingbirds wintering in dry tropical forest regions. Additionally, reduced habitat quality on breeding grounds due to widespread agrochemical exposure may further impact the species^28–30^. Ruby-throated hummingbirds are arriving to breeding grounds up to ${18}\,\text{d}$ earlier than they did historically in the northern parts of their range^38^. The substantive changes to the breeding phenology of ruby-throated hummingbirds during which the population increased and then declined suggests that a phenological mismatch with breeding surveys is unlikely to artefactually contribute to the apparent population decline.

In contrast to the general declines observed in the Selasphorus genus, Anna’s Hummingbirds have experienced an ecological release putatively associated with climate change and supplemental resource provisioning by humans^12,39^. The increase in carrying capacity for Anna’s hummingbirds in some Northern parts of their range are thought to be a result of increased nectar availability from feeders and eucalyptus trees, which bloom from October to April^40^, although eucalyptus availability would not apply in Canada. These additional resources alleviate the pressure of constricted nectar availability in winter months, when Anna’s hummingbirds begin their breeding season, therefore increasing the potential for a second brood^12,41^. Anna’s Hummingbirds may also possess physiological and behavioural adaptations not yet examined or quantified that allow them to tolerate the extreme cold weather in Canada.

Estimating species population trends and population sizes using BBS data has drawn criticism because surveys along routes could potentially fail to transect productive habitats, or underestimate populations^13,42,43^. Still, the current and rapidly developing Bayesian statistical methods used to analyze these data have matured considerably in the past half-century to extract ecologically important information from species in regions with low survey coverage^32,44–46^. Furthermore, these are the most rigorous methods available because it is possible to quantify uncertainties in trend estimates^47^. Advances in other broad-scale monitoring programs such as eBird will soon allow for a comparative data set on hummingbird population trends. Citizen-science tools such as eBird provide a powerful means for unstructured data collection because amateur enthusiasts contribute large quantities of opportunistic observational data on species occurrence or species abundance. Observers variably contribute other information relevant to the nature of the observation such as effort, time of observation, and duration of effort. Understanding of the current limitations of these tools is essential for developing accurate trend estimates. Monitoring programs combining citizen-science and BBS surveys have good potential to complement one another to identify and prioritize regions of conservation interest^48,49^ and provide more robust estimates over a broader region including the non-breeding grounds.

Hummingbird species across North America are clearly undergoing significant population changes. The steep declines reported and validated here among species in the *Selasphorus* genus are of particular concern to biodiversity conservation efforts. International conservation efforts such as the Partners in Flight organisation designate broad-tailed, rufous, and Allen’s hummingbirds as experiencing steep declines and under major threat throughout the species range^17^. Our detailed assessment of North American hummingbird species coupled with our validation analyses provide a critical framework in conservation science. Furthermore, the previously unreported declines of ruby-throated hummingbirds carry potentially important ecosystem-level consequences if left unaddressed, since this pollinator species alone fills it’s ecological niche in Eastern North America. We propose future studies using citizen-science data collection to target regions of high conservation priority for ruby-throated hummingbirds and species in the *Selasphorus* genus, followed by BBS analyses to monitor population trends in ongoing efforts to conserve these essential pollinators of North America. More detailed studies are also needed to investigate potential drivers of decline, particularly the impacts of agrochemicals throughout the annual cycle and habitat loss on the non-breeding grounds.

## Methods

### Breeding bird surveys

BBS surveys have been conducted since 1966 and involve $\sim 40\,{\text{km}}$ long transects consisting of 50 road-side point counts separated by $\sim 0.8\,{\text{km}}$^10^. Each route is surveyed by an expert once per year between late May and early July on fair weather days with the survey commencing approximately ${0.5}\,{\text{h}}$ before sunrise. All species and individuals detected within a ${400}\,{\text{m}}$ radius are recorded at each point count. For most analyses, abundance data is summed across all 50 points on each route to provide a single estimate of abundance per species per year. Data are excluded from statistical analyses when observations occur outside of permissible dates and times of day, or observations were collected during rain or high winds, according to USGS field procedures^50^. Our long-term continent-wide trend analyses included data from 59 to 2469 routes, and short-term trend analyses included data from 50 to 2123 routes. BBS route coverage was sufficient for analysis of 8 of the 14 commonly occurring hummingbird species in the USA and Canada^16^. The species that were excluded have ranges that fall primarily in Mexico with only small peripheral populations in southern regions of California, Arizona and Texas where there was insufficient BBS coverage. Data were considered insufficient when modelled strata did not comprise a minimum of three routes on which the modelled species was observed, a minimum of one such routes that was surveyed for 5 years or more, and a minimum of one route on which the species was observed in 3 years or more.

### Statistical analyses

Population trends and trajectories were estimated using a Bayesian hierarchical Generalized Additive Model with Year Effects (GAMYE)^32^ in R^51^. The survey-wide analyses were run during the annual analysis of the BBS data conducted by the Canadian Wildlife Service^52^, and additional summaries and maps were created with the R-package bbsBayes^53^. Data were stratified both by geopolitical regions (states and provinces), as well as internationally ratified Bird Conservation Regions (BCRs)^54^. Point estimates for trends are calculated as the median of the posterior distribution generated from Markov-chain Monte-Carlo methods. Credible intervals (CI) for parameter estimates are computed as percentiles of the posterior distribution of parameters. CIs are reported for the interval spanning 90% of the posterior distribution, unless otherwise stated. Trends described for long-term data include the period 1970, when systematic BBS surveying of hummingbirds began, to 2019. Trends described for short-term data include the period from 2009 to 2019.

To test the effect of survey date on species counts, we fit a modified BBS trend model. Peak number observations in the breeding season were allowed to vary by decade, incorporating an additional hierarchical generalized additive model structure to the standard GAMYE model. The smoothing function for this parameter was fit using a time-series approach, allowing each decade’s peak to lead or lag the preceding decade’s peak, allowing for the detection of a progressive change in peak observations through time. Scripts and data to run these analyses are provided in Availability of materials and data.

## Supplementary Information

Supplementary Information.

## Data Availability

BBS data and R scripts to analyze these data are provided by A.C.S. on Zenodo (DOI: 10.5281/zenodo.5273694). Data and R scripts for GAMYE model modifications accounting for survey-timing effects are also provided by A.C.S. on Zenodo (DOI: 10.5281/zenodo.5273572).
