# Supplementary material for: Current contrasting population trends among North American hummingbirds
Source: Sci Rep. 2021 Sep 15;11:18369. doi: 10.1038/s41598-021-97889-x (PMC8443710; doi:10.1038/s41598-021-97889-x)
Supplement: Supplementary file 1 — Supplementary Information. [file 41598_2021_97889_MOESM1_ESM.pdf]

I Supplementary Figures

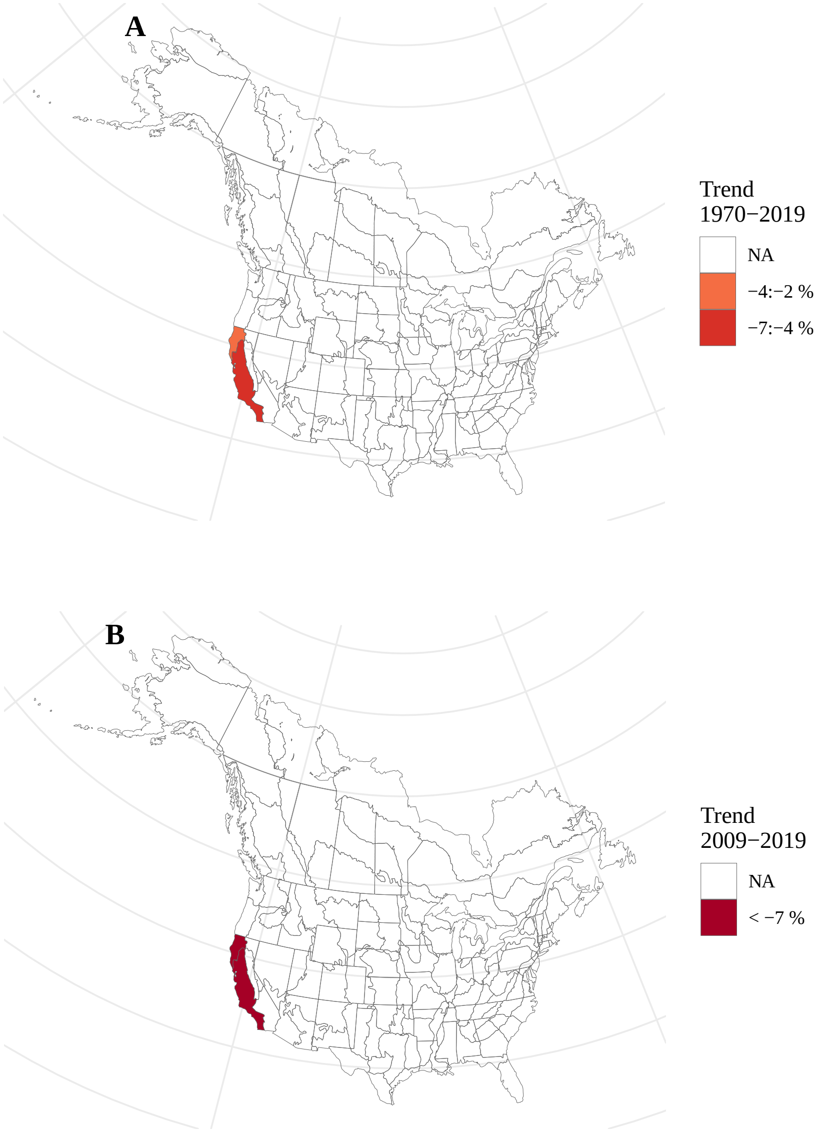

Figure S1. North American trend map of Allen's hummingbirds (*Selasphorus sasin*) over long-term modelling (A) and short-term modelling (B) analyses.

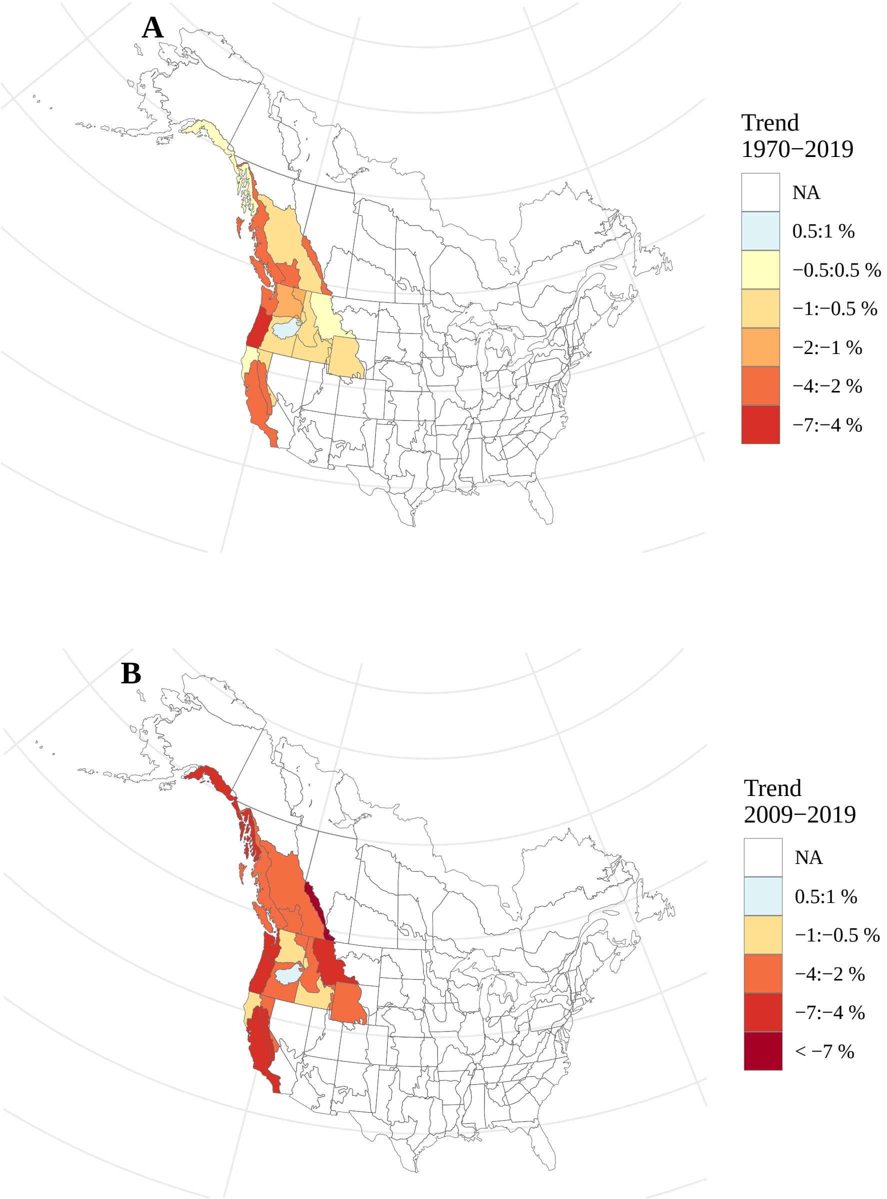

Figure S2. North American trend map of rufous hummingbirds (*Selasphorus rufus*) over long-term modelling (A) and short-term modelling (B) analyses.

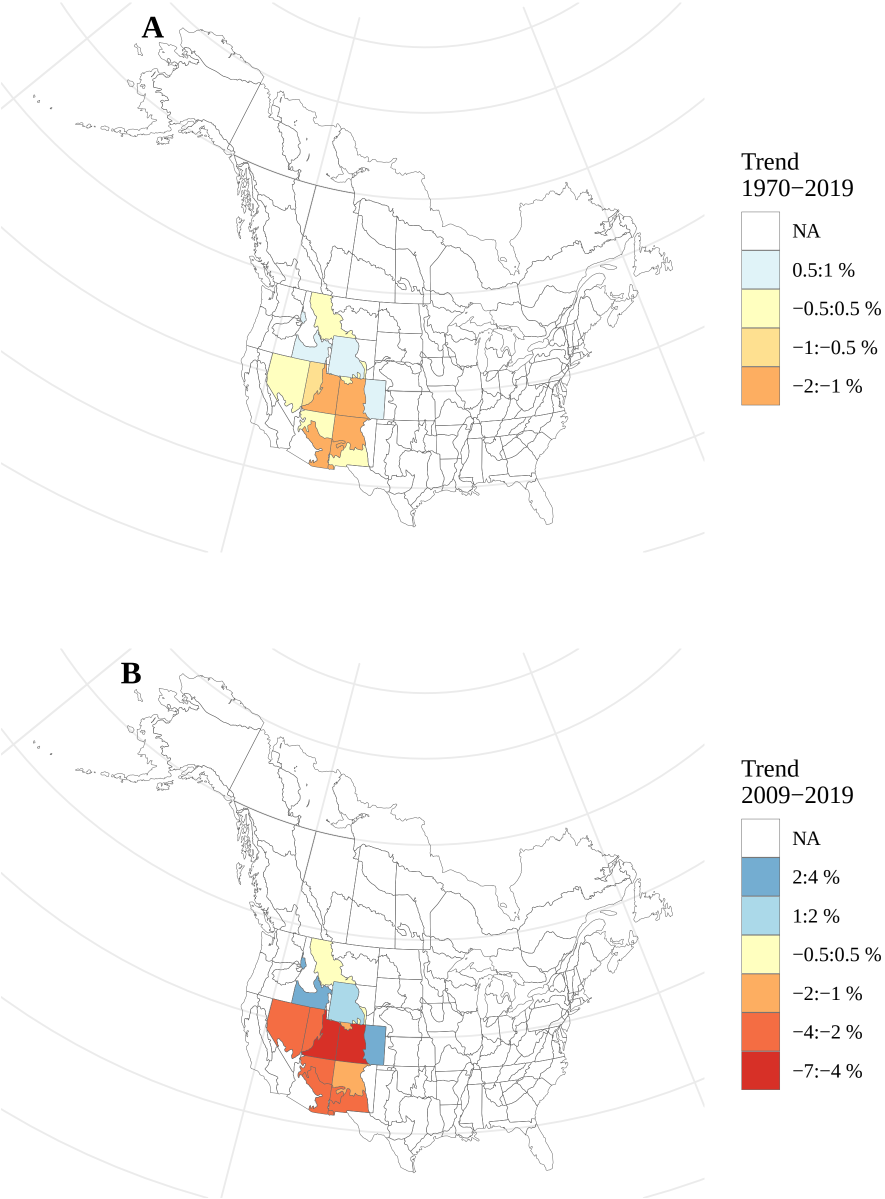

Figure S3. North American trend map of broad-tailed hummingbirds (*Selasphorus platycercus*) over long-term modelling (A) and short-term modelling (B) analyses.

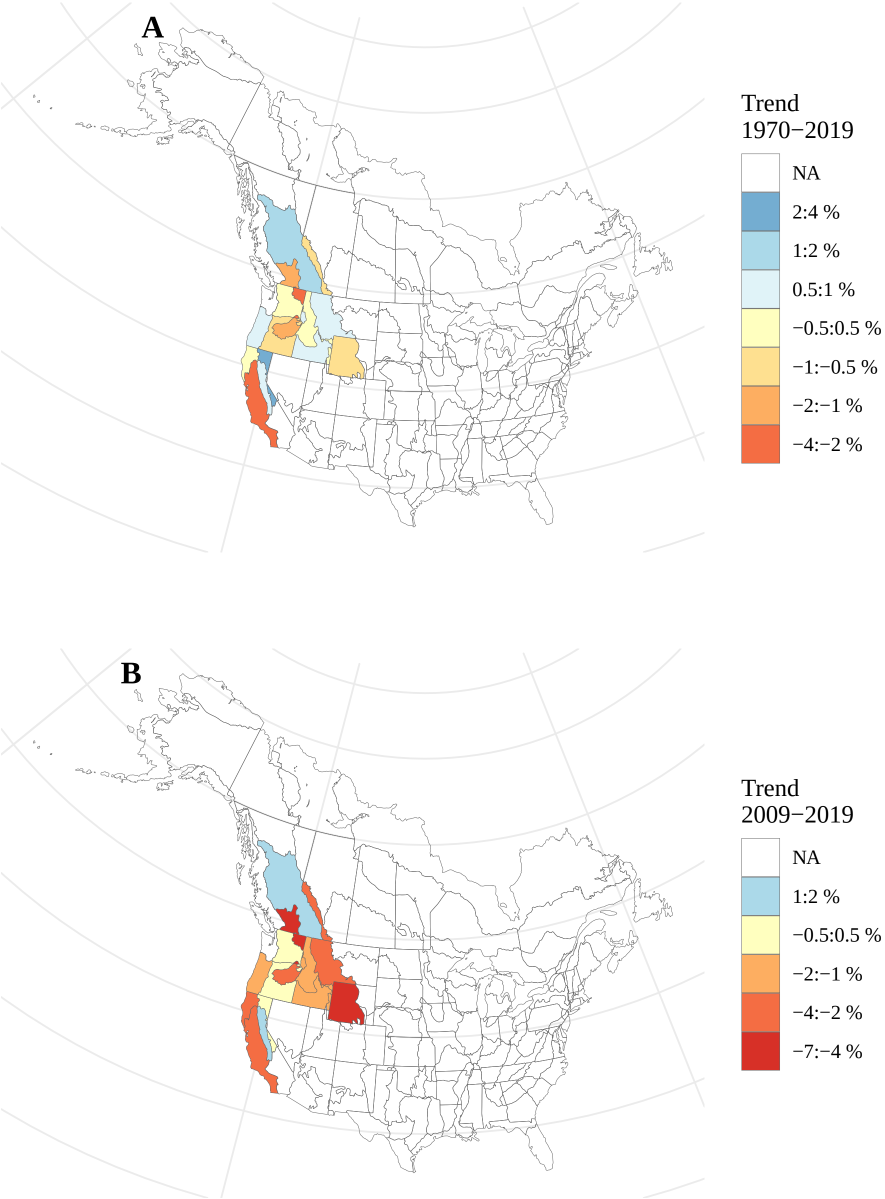

Figure S4. North American trend map of calliope hummingbirds (*Selasphorus calliope*) over long-term modelling (A) and short-term modelling (B) analyses.

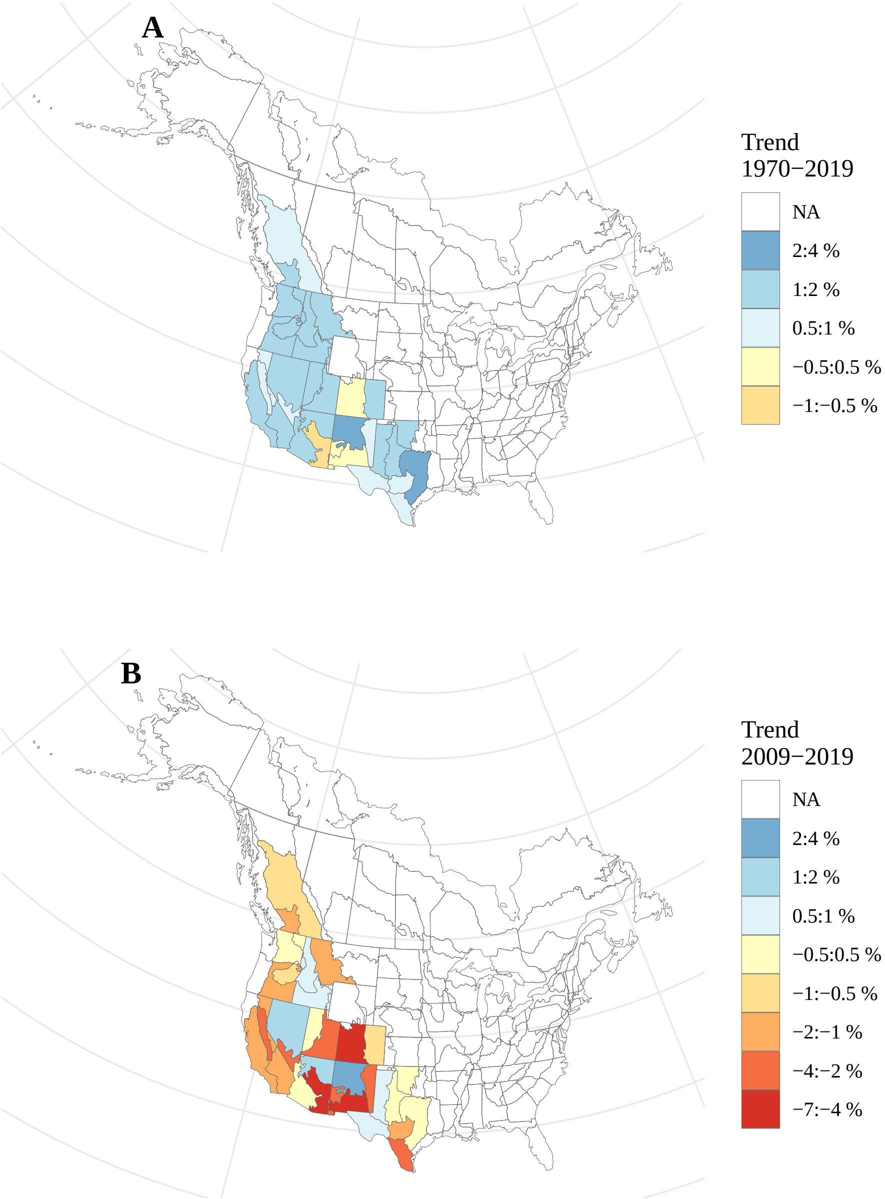

Figure S5. North American trend map of black-chinned hummingbirds (*Archilochus alexandri*) over long-term modelling (A) and short-term modelling (B) analyses.

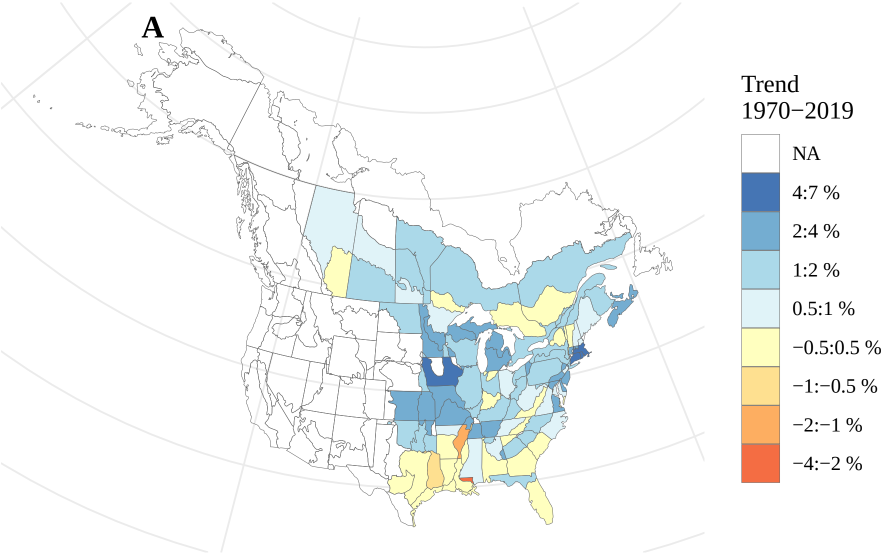

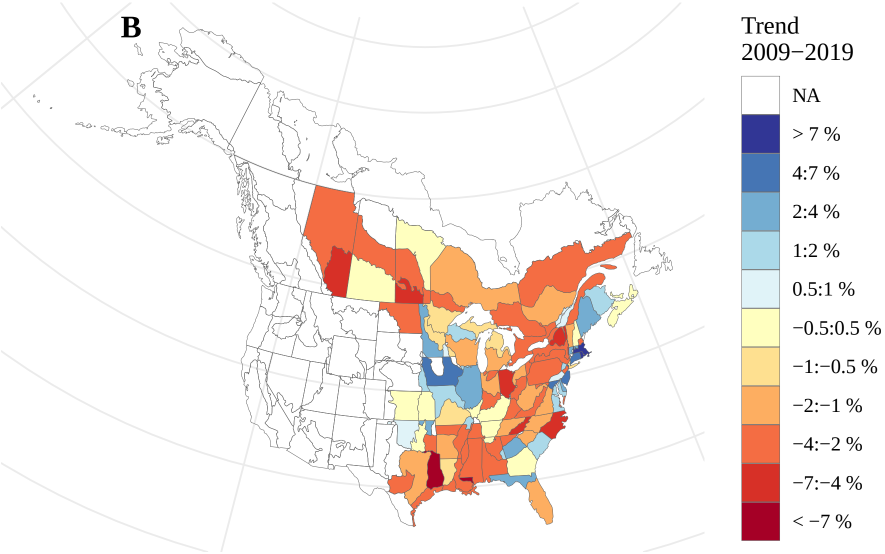

Figure S6. North American trend map of ruby-throated hummingbirds (*Archilochus colubris*) over long-term modelling (A) and short-term modelling (B) analyses.

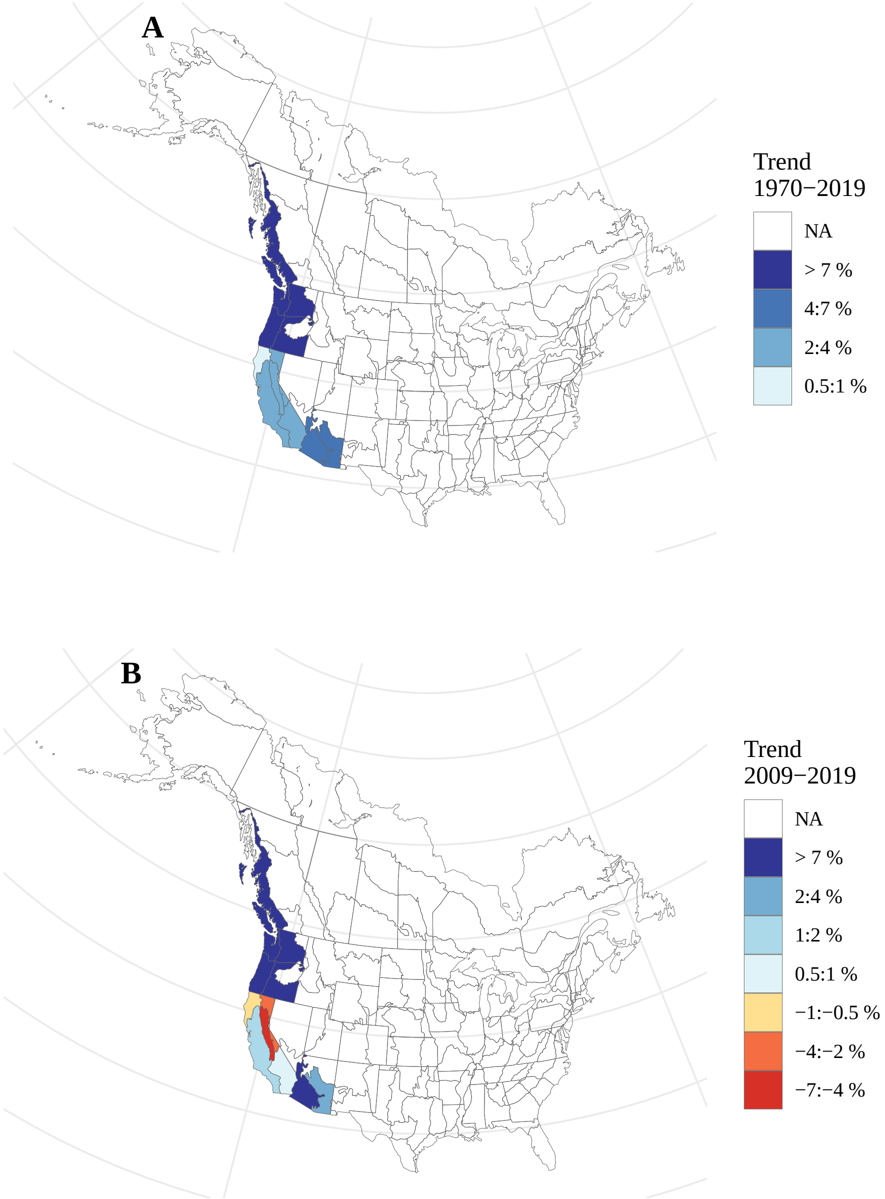

Figure S7. North American trend map of Anna's hummingbirds (*Calypte anna*) over long-term modelling (A) and short-term modelling (B) analyses.

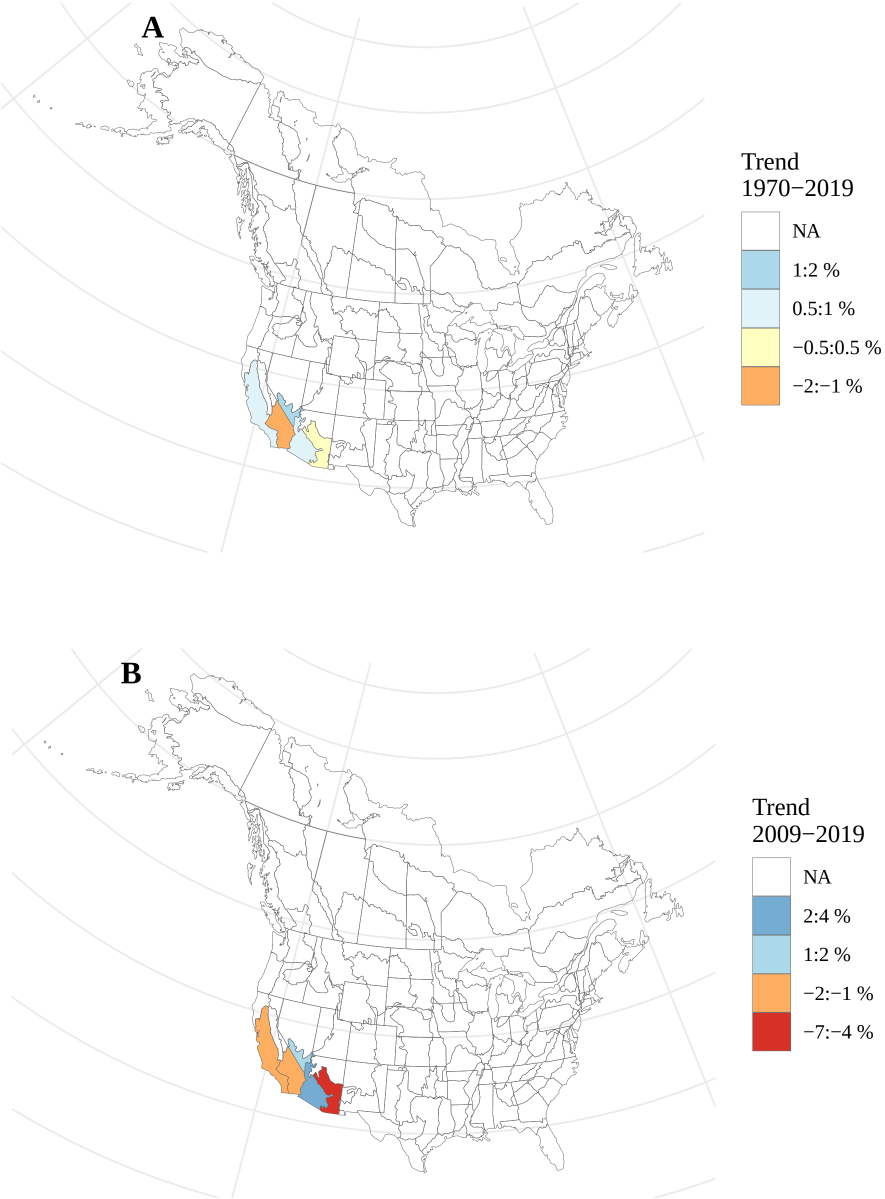

Figure S8. North American trend map of Costa's hummingbirds (*Calypte costae*) over long-term modelling (A) and short-term modelling (B) analyses.

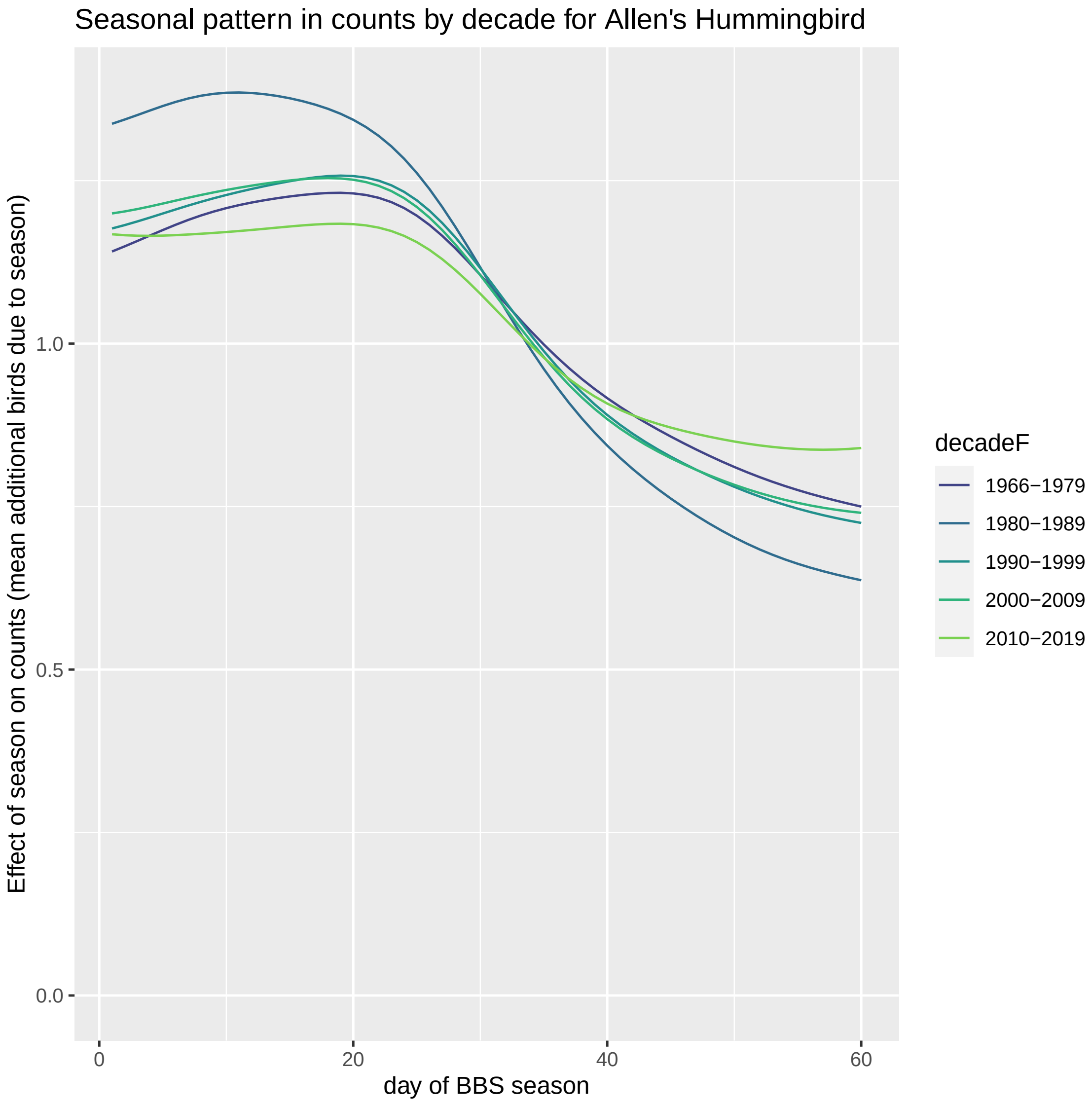

Figure S9. Effects of survey date on mean number of Allen's hummingbird (*S. sasin*) observations.

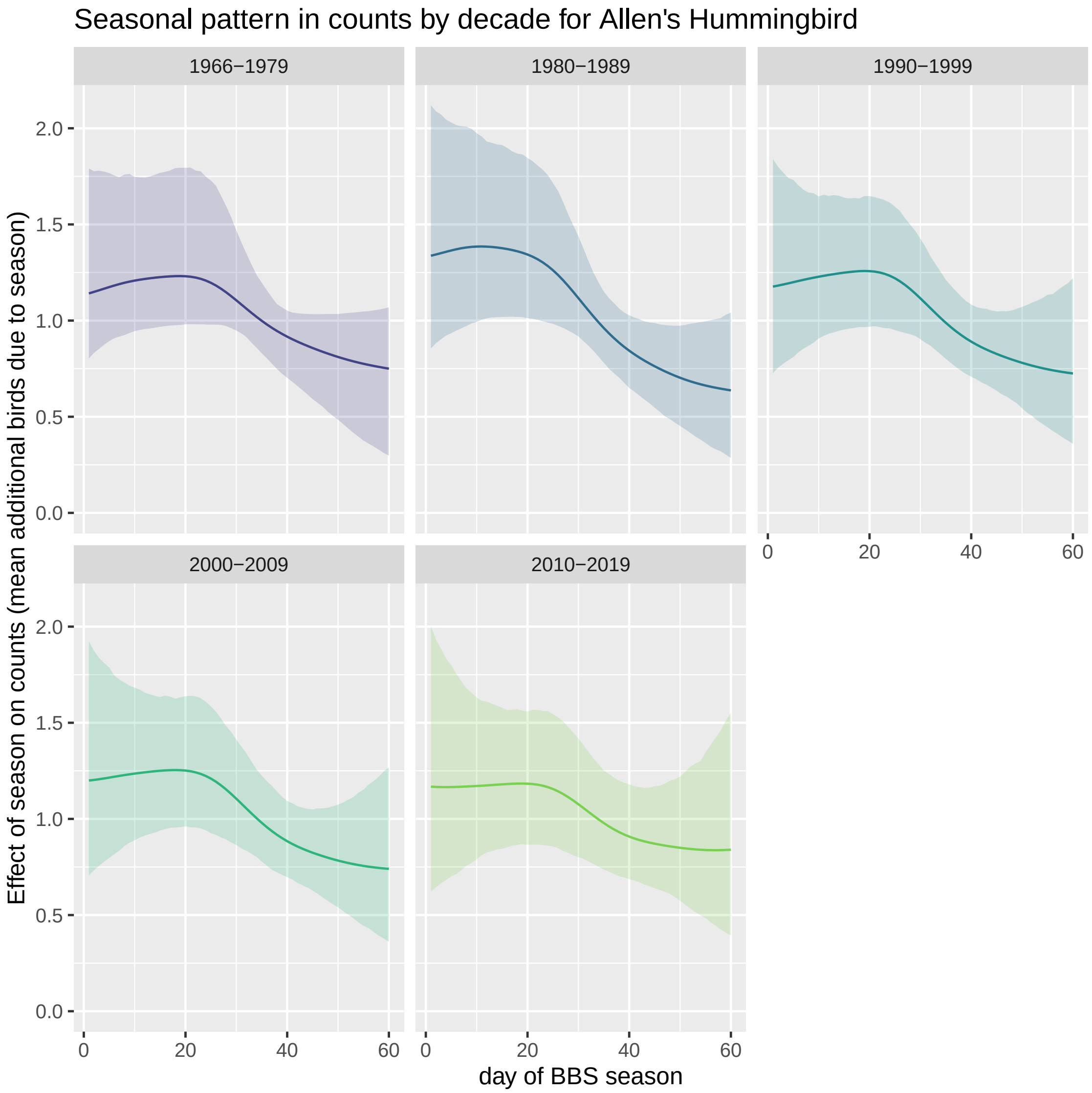

Figure S10. Effects of survey date on mean number of Allen's hummingbird (*S. sasin*) observations.

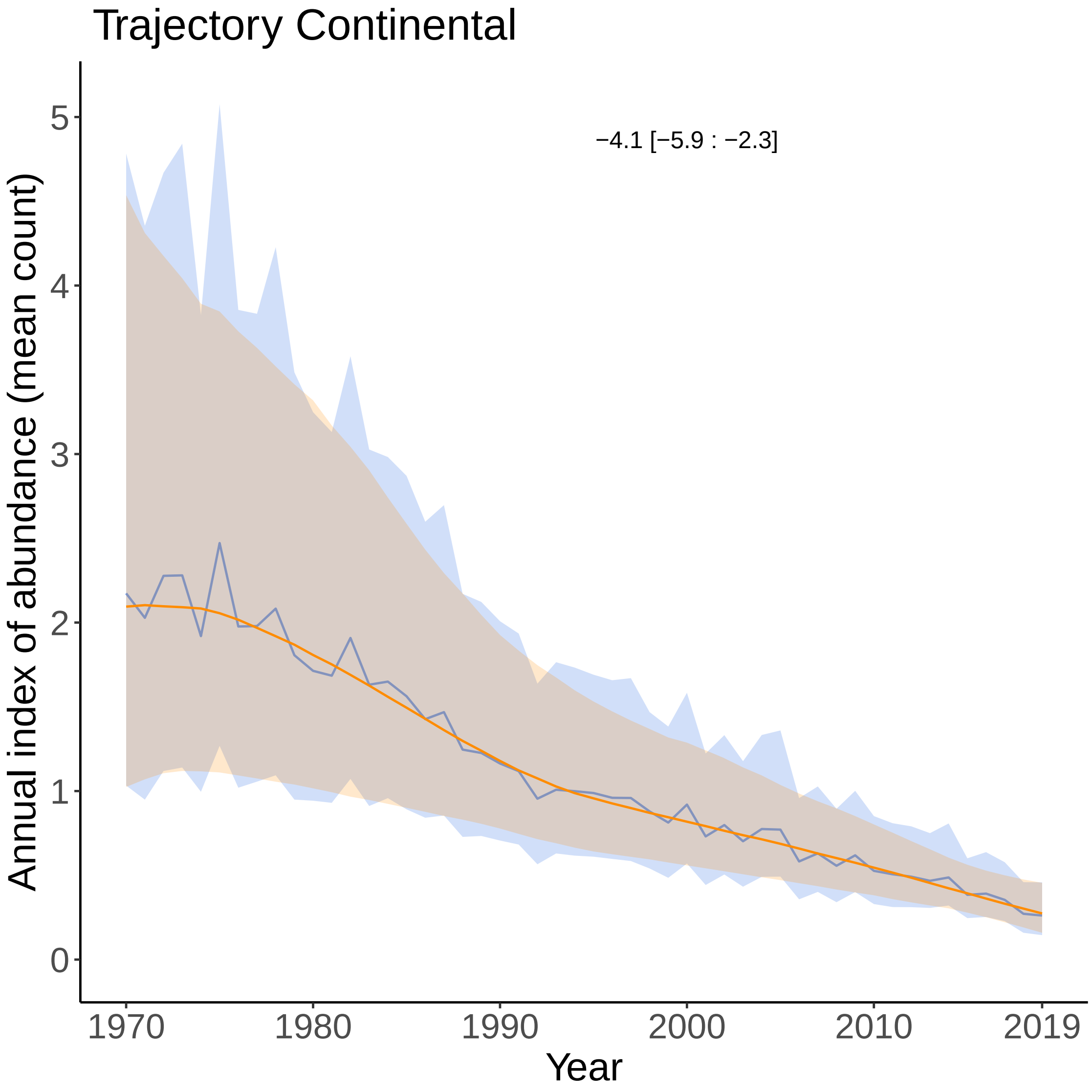

Figure S11. Long-term trend estimates for Allen's hummingbird (*S. sasin*) populations after accounting for effects of mismatch between surveys and species phenology.
